# Molecular Interplay between hypertension and dyslipidemia in cardiovascular pathogenesis: toward precision-based therapeutic strategies

**DOI:** 10.3389/fcvm.2026.1783567

**Published:** 2026-06-11

**Authors:** Boming Cao, Jingyi Pei, Ling Kong, Ying Han, Guangli Yan, Xijun Wang

**Affiliations:** 1 State Key Laboratory of Integration and Innovation of Classic Formula and Modern Chinese Medicine, National Chinmedomics Research Center, Metabolomics Laboratory, Department of Pharmaceutical Analysis, Heilongjiang University of Chinese Medicine, Harbin, China; 2 State Key Laboratory of Quality Research in Chinese Medicine, Macau University of Science and Technology, Taipa, Macao SAR, China

**Keywords:** cardiovascular disease, dyslipidemia, hypertension, inflammation, molecular mechanisms, targeted therapy

## Abstract

Cardiovascular disease (CVD) remains the foremost cause of global mortality. Hypertension and dyslipidemia, frequently coexisting, exert synergistic effects that substantially elevate CVD risk via interconnected molecular pathways. This review systematically examines the synergistic mechanisms underlying their combined contribution to CVD progression, with a focus on key pathways such as the RAAS/PPAR axis, AMPK/SIRT1 signaling, and NLRP3 inflammasome activation. We highlight how their interplay disrupts endothelial function, aggravates oxidative stress, and promotes chronic inflammation, thereby causing vascular damage and atherosclerosis. Furthermore, we examine emerging targeted therapies, including the combination of RAAS inhibitors with statins, PCSK9 inhibitors, as well as newer agents such as SGLT2 inhibitors and GLP-1 receptor agonists. These approaches represent promising multi-target strategies for improving clinical outcomes. Ultimately, this review underscores the need for a precision medicine framework that addresses the synergistic pathophysiology of hypertension and dyslipidemia, paving the way for personalized, pathway-integrated interventions aimed at restoring metabolic-vascular homeostasis rather than merely treating disease.

## GRAPHICAL ABSTRACT

**Figure:**
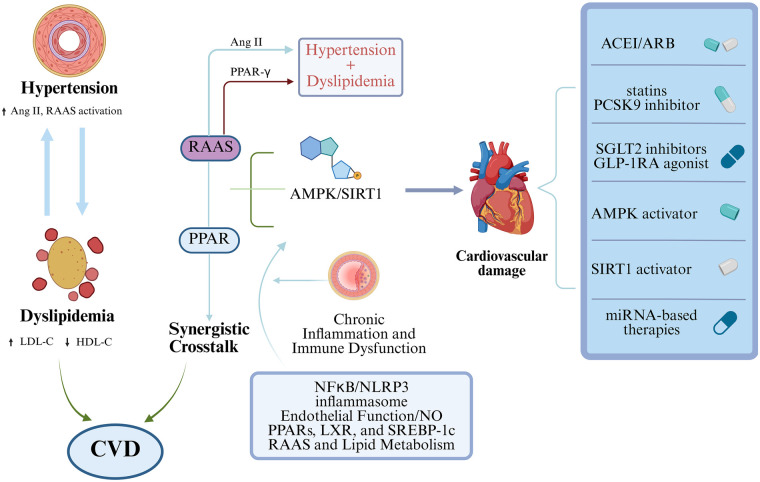
This figure summarizes the synergistic mechanisms and targetable pathways by which hypertension and dyslipidemia contribute to cardiovascular disease, and highlights potential therapeutic targets for precision medicine.

## Highlights

- Uncovers the multi-pathway molecular network through which hypertension and dyslipidemia synergistically accelerate cardiovascular diseases.
- Advocates a paradigm shift in cardiovascular disease management from single-target pharmacotherapy to multi-target restoration of metabolic-vascular homeostasis.

## 1 Introduction

Cardiovascular disease (CVD) is the leading cause of death and disability worldwide (1). According to World Health Organization (WHO) statistics, CVD was responsible for approximately 19.8 million deaths in 2022, representing 32% of all deaths worldwide. Over three-quarters of CVD deaths take place in low- and middle-income countries, imposing a severe burden on both public health and social resources (2). The pathogenesis of CVD is complex, involving multiple pathological mechanisms including atherosclerosis, endothelial dysfunction, chronic inflammation, oxidative stress, lipid deposition, and myocardial remodeling (3, 4). Current clinical management primarily relies on pharmacological interventions targeting these pathways, including antihypertensive, lipid-lowering, antiplatelet medications, and anti-inflammatory agents (5–8). However, single-target therapies often yield suboptimal efficacy and are frequently limited by side effects, drug resistance, and poor long-term adherence. This highlights an urgent need for more integrated therapeutic strategies that address the complex pathophysiology of CVD.

Among numerous risk factors for CVD, hypertension and dyslipidemia are two of the most prevalent and potent drivers of disease progression. Hypertension promotes vascular stiffening and cardiac overload by activating the renin-angiotensin-aldosterone system (RAAS), inducing vascular endothelial dysfunction, and triggering oxidative stress (9, 10). Dyslipidemia, particularly elevated low-density lipoprotein cholesterol (LDL-C) and reduced high-density lipoprotein cholesterol (HDL-C), further damages blood vessels by accelerating atherosclerosis and activating inflammatory and immune pathways (11–13). More critically, both clinical and basic research demonstrate that these conditions do not exist in isolation. Instead, they form a positive feedback loop via multi-level, multi-pathway molecular crosstalk, synergistically amplifying vascular inflammation, metabolic dysregulation, and myocardial injury, thereby multiplicatively increasing the risk of cardiovascular events (14–16).

Therefore, examining the pathophysiological mechanisms of CVD from the perspective of “hypertension and dyslipidemia interplay” offers significant scientific and clinical insight. This review aims to systematically elucidate the synergistic pathogenic mechanism through which hypertension and dyslipidemia co-drive CVD progression, with a particular focus on key interactive pathways such as the RAAS/PPAR axis, AMPK/SIRT1 energy metabolism signaling, and NLRP3 inflammasome activation. We also explore the translational potential of combined therapeutic strategies, such as RAAS inhibitors paired with statins, PCSK9 inhibitors, or SGLT2 inhibitors, which have been shown to synergistically improve hemodynamic and lipid profiles while more effectively reducing cardiovascular event risk (17, 18).

In summary, this review synthesizes current evidence on the collaborative role of hypertension and dyslipidemia in CVD pathogenesis, emphasizing molecular networks, regulatory crosstalk, and advances in multi-targeted therapies. It seeks to inform a precision medicine approach that shifts the paradigm from single-disease management toward integrated, pathophysiology-based intervention for cardiovascular comorbidity.

## 2 Core pathogenesis of CVD

**Figure 1:**
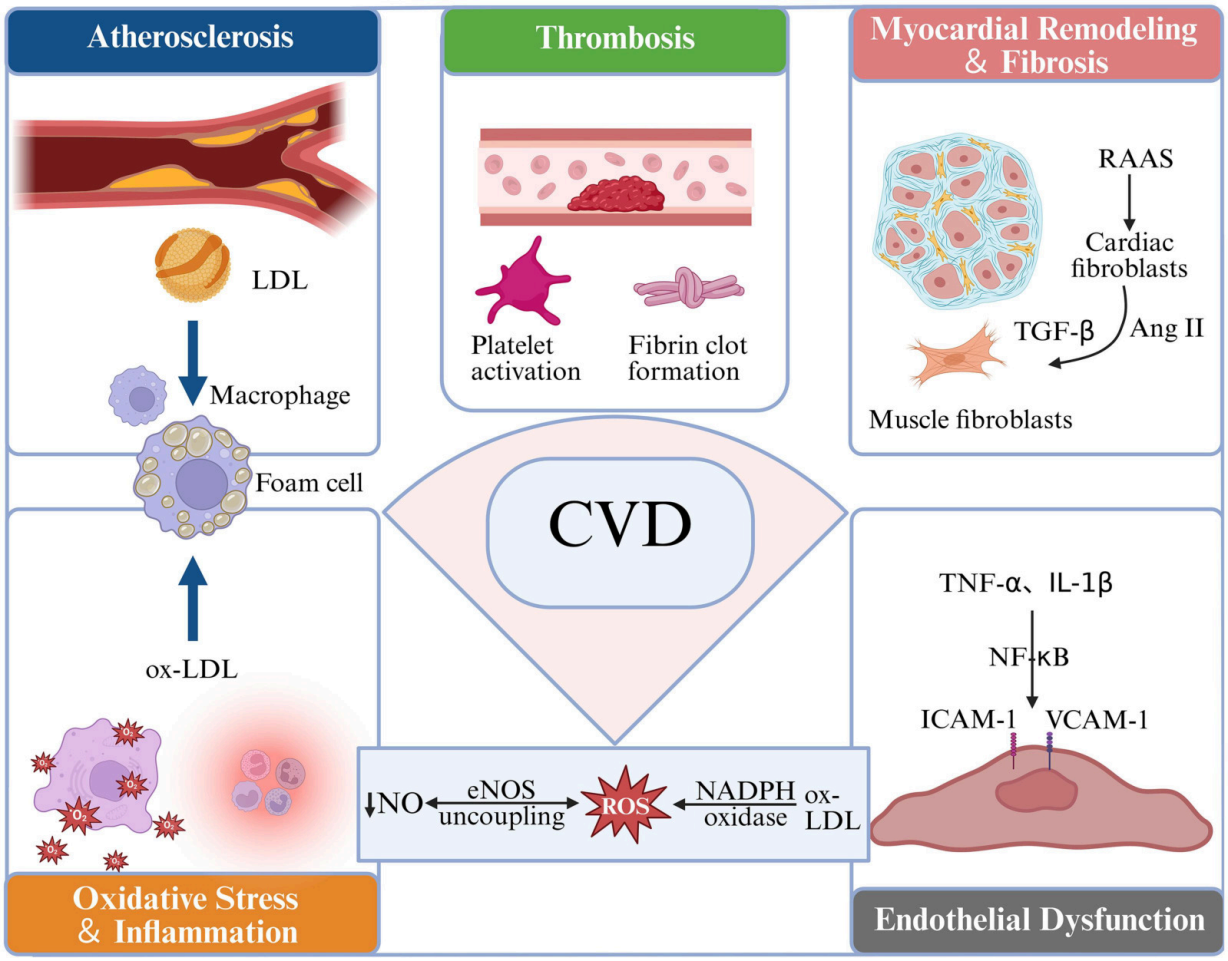
The core pathogenesis of CVD includes atherosclerosis, thrombosis, endothelial dysfunction, oxidative stress and inflammation, myocardial remodeling and fibrosis and other key pathological processes.

The core pathophysiology of CVD involves several interconnected mechanisms, such as atherosclerosis, thrombosis, endothelial dysfunction, oxidative stress and inflammation, myocardial remodeling, and fibrosis. These processes interact dynamically, collectively driving the onset and progression of CVD and forming a multi-layered pathological network that extends from initial vascular injury to end-organ damage (Figure 1). It is worth noting that vascular dysfunction serves as a key pathological link between hypertension, dyslipidemia, and the progression of cardiovascular disease. Oxidative stress, inflammation, and lipid toxicity associated with hypertension and dyslipidemia collectively damage endothelial cells and vascular smooth muscle cells (VSMCs), thereby promoting vascular remodeling, atherosclerosis, fibrosis, and thrombosis.

### 2.1 Atherosclerosis

Atherosclerosis, which is the primary pathological basis of CVD, involves a complex process driven by multiple mechanisms. Its core pathways include abnormal lipid deposition, chronic inflammatory responses, and structural remodeling of the vascular wall. In dyslipidemia, LDL-C accumulates beneath the endothelium and undergoes oxidative modification to form oxidized LDL (ox-LDL). Ox-LDL promotes endothelial dysfunction, induces monocyte chemotaxis, and facilitates macrophage lipid uptake via scavenger receptors, leading to foam-cell formation and the initiation of atherosclerotic plaques (19, 20). Hypertension further accelerates this process by increasing vascular wall shear stress and mechanical injury, which aggravates endothelial dysfunction and enhances vascular permeability, thereby facilitating LDL infiltration and oxidation. Mechanistically, hypertension- and dyslipidemia-associated oxidative stress and inflammation act synergistically to activate VSMCs. Under these stimuli, VSMCs undergo phenotypic switching, proliferation, and migration, contributing to plaque progression, vascular stiffening, fibrosis, and impaired plaque stability. As plaques progress, rupture or erosion may expose prothrombotic substances, triggering platelet aggregation and thrombus formation, ultimately leading to acute coronary syndromes, ischemic stroke, and other adverse cardiovascular events (21, 22). Current clinical intervention strategies primarily include statins, which significantly reduce LDL-C levels by inhibiting HMG-CoA reductase. In addition, PCSK9 inhibitors further enhance LDL receptor activity, substantially lowering residual cardiovascular risk (23). The combined use of GLP-1 receptor agonists and SGLT2 inhibitors is also clinically feasible, having demonstrated multiple beneficial effects, including anti-inflammatory properties and plaque stabilization. This combination strategy offers a novel approach for the comprehensive management of atherosclerosis (24).

### 2.2 Thrombosis

Thrombosis, a direct contributor to CVD events including myocardial infarction and ischemic stroke, involves three key pathological mechanisms: vascular endothelial injury, platelet activation, and aberrant activation of the coagulation system. In the context of atherosclerosis, plaque rupture or erosion exposes subendothelial collagen and tissue factor, activating platelets and promoting their adhesion and aggregation to form white thrombi. Concurrently, the tissue factor pathway initiates the extrinsic coagulation cascade, leading to fibrin network formation and red thrombus expansion (22, 25). Furthermore, certain blood flow disturbances, including high shear stress induced by hypertension, can further enhance platelet activation and coagulation factor aggregation (26). Inflammatory mediators (e.g., IL-6 and TNF-α) inhibit the fibrinolytic system through the upregulation of plasminogen activator inhibitor-1 (PAI-1), which stabilizes thrombi and extends their lifespan (27). Traditionally, treatment has primarily relied on antiplatelet agents (e.g., aspirin and P2Y12 inhibitors) (28) and anticoagulants (e.g., warfarin), but these carry a risk of bleeding. In recent years, direct-acting oral anticoagulants (DOACs) have demonstrated efficacy comparable to or superior to warfarin in preventing stroke among patients with atrial fibrillation. They also offer greater safety advantages, particularly by significantly reducing the risk of intracranial hemorrhage, leading to their increasingly widespread clinical adoption (29).

### 2.3 Endothelial dysfunction

Endothelial dysfunction represents a critical early event in CVD development, characterized by reduced nitric oxide (NO) bioavailability, impaired vasoregulatory function, and a heightened proinflammatory state. Under normal conditions, endothelial nitric oxide synthase (eNOS) is expressed in vascular endothelial cells and catalyzes the conversion of L-arginine into NO, and reduces leukocyte adhesion to the endothelium (30). Under the influence of risk factors, including hypertension and dyslipidemia, ox-LDL binds to the LOX-1 receptor to activate nicotinamide adenine dinucleotide phosphate (NADPH) oxidase. This enzyme subsequently generates reactive oxygen species (ROS), which in turn induce eNOS uncoupling. Although eNOS normally generates NO, its uncoupling causes a functional switch, leading the enzyme to produce superoxide anions instead. The increased generation of superoxide anions subsequently aggravates oxidative stress (19). Concurrently, inflammatory mediators (e.g., TNF-α, IL-1β) upregulate adhesion molecules (VCAM-1, ICAM-1) and chemokine expression via the nuclear factor kappa B (NF-κB) pathway, promoting monocyte infiltration and intimal accumulation (31). Endothelial cells also enhance vasoconstriction and remodeling by releasing endothelin-1 (ET-1), forming a positive feedback loop. Collectively, these alterations lead to increased vascular tension, heightened permeability, and the initiation of atherosclerosis (32). Several strategies aim to improve endothelial function. These include RAAS inhibitors, statins, SGLT2 inhibitors, and antioxidants. They work through multiple mechanisms to restore NO homeostasis and suppress oxidative and inflammatory responses, thereby helping to delay the progression of CVD. Among these, the benefits of RAAS inhibitors have been validated in clinical studies. For example, ramipril significantly improves endothelial function and reduces inflammatory biomarker levels in children undergoing dialysis (33).

### 2.4 Oxidative stress and inflammation

Oxidative stress and inflammation interact closely in the onset and progression of CVD, jointly constituting its core pathological mechanism. Oxidative stress refers to a state where excessive ROS production overwhelms the body's antioxidant defense system. Excessive ROS can directly damage vascular endothelial cells, triggering oxidative modifications of lipids, proteins, and DNA. This leads to endothelial dysfunction, diminished vasodilatory capacity, and reduced bioavailability of NO (34). These alterations promote vasoconstriction, smooth muscle cell proliferation, and migration, laying the groundwork for atherosclerosis. Concurrently, oxidatively modified ox-LDL can be phagocytosed by macrophages to form foam cells, further stimulating inflammatory responses, which in turn amplify and sustain the inflammation (11). Oxidative stress activates signaling pathways such as NF-κB and NLRP3 inflammasome, inducing the release of multiple inflammatory mediators (e.g., IL-1β, IL-6, and TNF-α) and causing chronic inflammatory infiltration of the vascular wall (35). Inflammation further increases ROS production by activating NADPH oxidases (e.g., NOX1) and inducing mitochondrial dysfunction, thereby creating a vicious cycle (36). Additionally, endothelial cell activation and enhanced expression of adhesion molecules promote monocyte adhesion and infiltration, accelerating plaque formation and instability (37). Antioxidants (e.g., NAC) and anti-inflammatory therapies (e.g., IL-1β antibodies) demonstrate cardiovascular protective effects in experimental models, highlighting the therapeutic potential of targeting this pathway in CVD management (38, 39).

### 2.5 Myocardial remodeling and fibrosis

Cardiac remodeling and fibrosis constitute key pathological foundations for CVD progression, triggered by various stimuli ranging from hypertension, myocardial infarction, and metabolic abnormalities. In hypertension, prolonged mechanical overload and neurohumoral activation, particularly activation of the renin-angiotensin-aldosterone system and sympathetic nervous system, promote cardiomyocyte hypertrophy, apoptosis, and abnormal extracellular matrix (ECM) deposition (40, 41). Angiotensin II and transforming growth factor-β (TGF-β) are central profibrotic mediators that activate myocardial fibroblasts and induce their differentiation into myofibroblasts. Activated myofibroblasts produce excessive collagen I and III, leading to interstitial fibrosis and increased myocardial stiffness (42). In parallel, metabolic abnormalities such as dyslipidemia further aggravate myocardial remodeling by promoting oxidative stress, inflammatory responses, mitochondrial dysfunction, and fibrotic signaling, thereby altering the myocardial microenvironment (43). These structural changes not only impair myocardial elasticity but also contribute to electrical remodeling and systolic and diastolic dysfunction, increasing the risk of heart failure and arrhythmias (44). Therapeutically, RAAS inhibitors, including angiotensin-converting enzyme inhibitors (ACEIs) and angiotensin receptor blockers (ARBs), and aldosterone receptor antagonists (e.g., spironolactone) serve as first-line agents to delay myocardial remodeling (45). β-blockers help reduce myocardial fibrosis by suppressing sympathetic nerve activity, decreasing myocardial oxygen consumption, and indirectly inhibiting the abnormal activation of the renin-angiotensin-aldosterone system (46).

## 3 The synergistic pathogenic mechanism of hypertension and dyslipidemia

**Figure 2:**
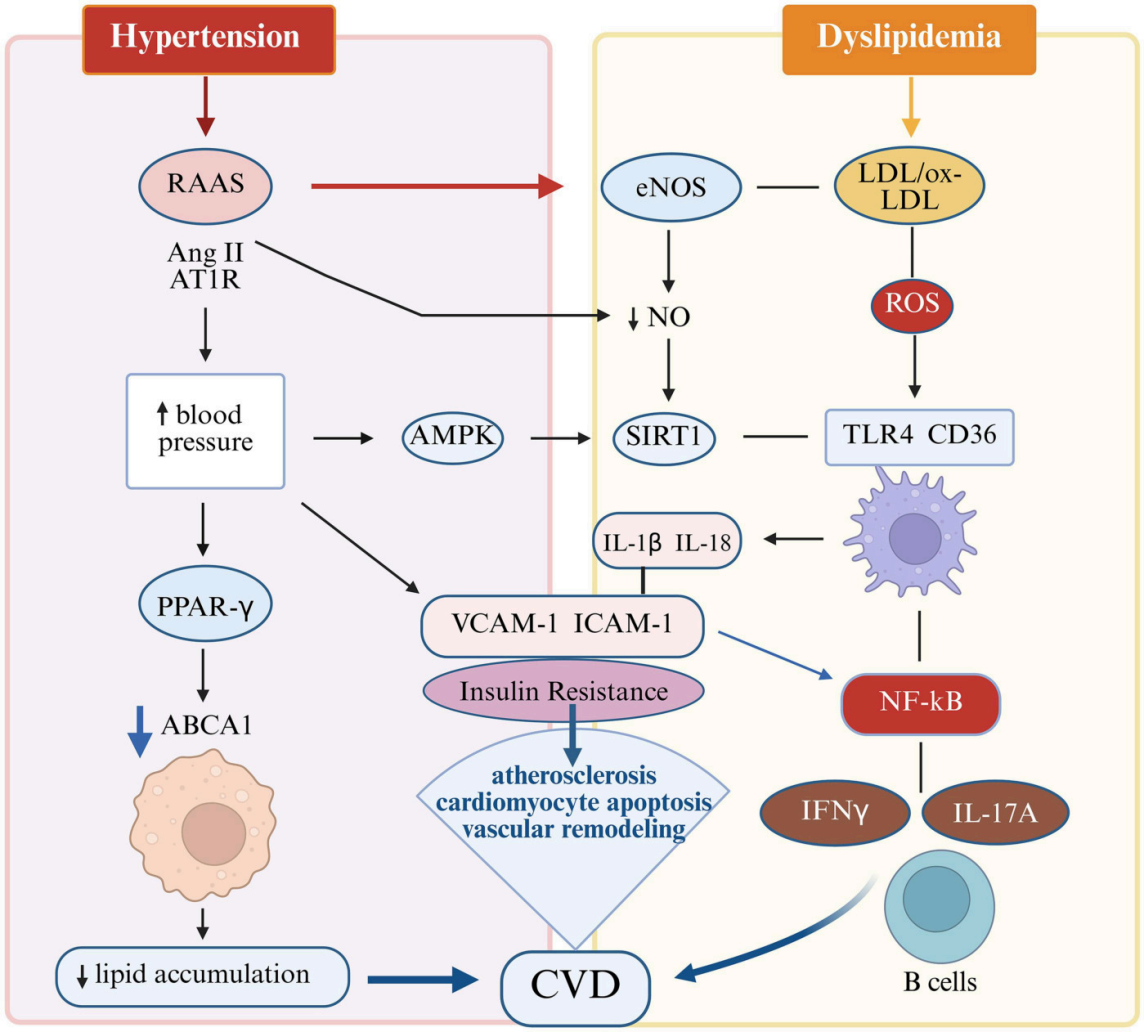
The synergistic pathogenic mechanism of hypertension and dyslipidemia on cardiovascular events.

Hypertension and dyslipidemia, which are the two core risk factors for CVD, are often co-morbid and significantly amplify CVD risk through a complex network of molecular interactions. A systematic elucidation of the synergistic mechanism of hypertension and dyslipidemia in the development of CVD will provide a theoretical basis for the exploration of targeted therapeutic strategies, and therefore promote the paradigm shift from single-disease management to synergistic intervention for co-morbidities. This section summarizes how hypertension and dyslipidemia form a positive feedback loop to accelerate vascular injury and end-organ disease from three perspectives: signaling pathway interactions, metabolic stress and mitochondrial dysfunction, and chronic inflammation and immune abnormalities (Figure 2). The pathological mechanisms described in Section 2 can also be interpreted within the context of hypertension-dyslipidemia interaction, as both conditions synergistically contribute to endothelial dysfunction, oxidative stress, inflammation, and vascular remodeling, thereby accelerating CVD progression.

### 3.1 Interactions and regulatory networks of signaling pathways

In the synergistic interaction between hypertension and dyslipidemia, the interplay of signaling pathways constitutes a core mechanism driving disease progression. Significant cross-regulatory relationships exist between the RAAS and the peroxisome proliferator-activated receptor (PPAR) signaling pathway. Hypertension activates the RAAS, which not only elevates blood pressure but also inhibits PPAR-*γ* expression, thereby impairing ABCA1-mediated cholesterol efflux and aggravating dyslipidemia (47). In contrast, PPAR agonists can suppress RAAS activity while improving endothelial function and lipid metabolism (48). Additionally, the eNOS-NO pathway plays a pivotal role in this synergistic mechanism. Hypertension suppresses eNOS activity through oxidative stress and eNOS uncoupling, thereby reducing NO bioavailability and impairing vasodilation. Dyslipidemia-derived ox-LDL further aggravates endothelial dysfunction by enhancing ROS production and inhibiting eNOS signaling (30). Moreover, Insulin Resistance (IR) is increasingly recognized as an important pathophysiological link connecting hypertension, dyslipidemia, and CVD progression. IR contributes to endothelial dysfunction, oxidative stress, inflammation, and vascular remodeling, while dyslipidemia-derived lipotoxicity further aggravates insulin signaling impairment. Together, these processes form a positive feedback loop that promotes atherosclerosis, vascular remodeling, and CVD progression (49, 50). Concurrently, reduced HDL levels and elevated LDL/ox-LDL ratios further aggravate endothelial dysfunction and vascular remodeling, forming a positive feedback loop that accelerates CVD progression.

### 3.2 Metabolic stress and mitochondrial dysfunction

In the pathological state where hypertension coexists with dyslipidemia, metabolic stress and mitochondrial dysfunction form the core components of their synergistic pathogenic mechanism. On one hand, adenosine monophosphate-activated protein kinase (AMPK), a key sensor of intracellular energy status, exhibits significantly suppressed activity in the hypertensive environment. This inhibition impairs fatty acid oxidation and glucose metabolism, leading to cellular energy deficiency and metabolic disorders (51). On the other hand, Silent Information Regulator 1 (SIRT1), which is an NAD⁺-dependent deacetylase, plays a crucial role in mitochondrial biogenesis, antioxidant responses, and cell lifespan regulation. However, in cases of hypertension complicated by dyslipidemia, both the expression and activity of SIRT1 decline, leading to a further weakening of the cell's antioxidant defense mechanisms (52). Additionally, hypertension can induce mitochondrial stress, promoting excessive production of reactive ROS, damaging mitochondrial membrane structures, and forming a vicious cycle between oxidative stress and dysfunction (53). With the intensification of lipid toxicity, cardiomyocytes undergo apoptosis, experience impaired ATP synthesis, and develop myocardial structural remodeling. Ultimately, through a cascade of metabolic and structural imbalances, these factors collectively drive CVD toward end-stage conditions like cardiomyopathy and atherosclerosis.

### 3.3 Synergistic amplification of chronic inflammation activation and immune abnormalities

In the context of hypertension and dyslipidemia synergistically promoting CVD development, the synergistic amplification effect between chronic inflammation activation and immune dysregulation is considered one of the core pathological mechanisms. Hypertension induces endothelial dysfunction by increasing blood flow shear stress and vascular wall tension, thereby upregulating the expression of adhesion molecules (e.g., VCAM-1 and ICAM-1) and promoting monocyte migration into the vascular endothelium (54). Meanwhile, dyslipidemia, particularly elevated LDL-C levels and the accumulation of its oxidized product ox-LDL, allows these lipids to be recognized and internalized via TLR4 and CD36 receptors on macrophage surfaces. This process activates the NLRP3 inflammasome, leading to the release of proinflammatory cytokines such as IL-1β and IL-18 (55, 56). These two mechanisms act synergistically to sustain the activation of key inflammatory pathways like NF-κB, thus establishing a low-grade, systemic chronic inflammatory state. Furthermore, the adaptive immune system actively participates in this process: hypertension and dyslipidemia jointly promote the differentiation of CD4⁺ T cells toward Th1 and Th17 subtypes, enhancing the expression of IFN-*γ* and IL-17A, which further exacerbates local and systemic vascular inflammatory responses. B cells have also been demonstrated to participate in lipid antigen presentation and autoantibody production, thereby amplifying the immune response (57, 58). Chronic inflammation and immune imbalance interact via the aforementioned mechanisms, forming a positive feedback loop. This loop continuously drives the progression of atherosclerosis, along with vascular remodeling and plaque instability. In turn, these changes significantly increase the risk of acute cardiovascular events.

## 4 Key molecular pathways and targets

**Figure 3:**
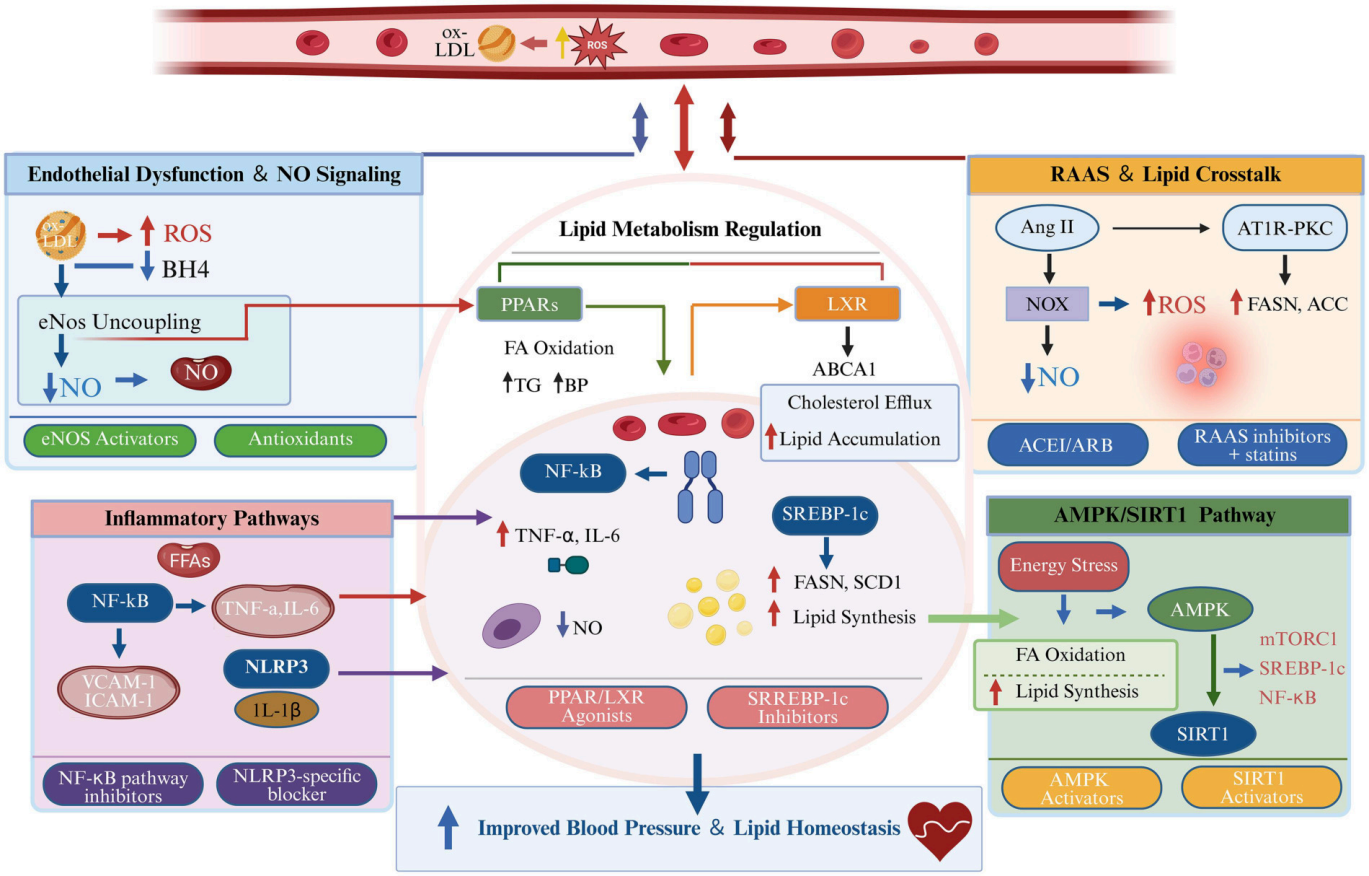
The integrated molecular mechanisms, targeted pathways, and therapeutic targets between dyslipidemia and hypertension.

The understanding of the synergistic mechanism at the molecular level lays the theoretical foundation for targeted therapy, which is the core of the logical progression from “mechanism interpretation” to “precision intervention”. This section systematizes the specific molecular pathways and regulatory nodes involved in the synergistic pathogenesis of hypertension and dyslipidemia. Key signaling pathways, such as endothelial function and nitric oxide signaling, inflammatory pathways (NF-κB and NLRP3 inflammasome), lipid metabolism regulatory networks (PPARs, LXRs, SREBP-1c), the interactions between RAAS and lipid metabolism, and the AMPK/SIRT1 signaling pathway, are elaborated in the regulation of synergistic pathogenesis of hypertension and dyslipidemia (Figure 3) (Table 1).

**Table 1:** Key molecular pathways and targeted therapeutic strategies in the synergistic interaction between hypertension and dyslipidemia.

Key pathways/mechanisms	Core target/molecule	Role in synergistic pathogenesis	Corresponding therapeutic strategies and drugs
Endothelial function and the NO signaling pathway	eNOS, NO, ox-LDL, BH4	eNOS uncoupling and reduced NO bioavailability lead to vasodilatory dysfunction, oxidative stress, and inflammation.	Drugs to improve endothelial function: SGLT2 inhibitors (to promote NO bioavailability). Antioxidants: as potential adjunctive therapeutic strategies.
Inflammatory signaling pathways (NF-κB, NLRP3 inflammasome)	NF-κB, NLRP3, IL-1β, IL-18, TNF-α	Mediates chronic low-grade inflammation and promotes inflammatory cell infiltration, endothelial dysfunction, and plaque instability in the vessel wall.	Specific inflammatory pathway inhibitors: NLRP3 inhibitors (e.g., MCC950), NF-κB inhibitors (e.g., Bay 11-7082). Drugs with anti-inflammatory effects: GLP-1 receptor agonists (inhibit NF-κB).
Lipid metabolism regulatory pathways (PPARs, LXR, SREBP-1c)	PPARα/*γ*, LXR, SREBP-1c, ABCA1	Regulates dyslipidemia, reverse cholesterol transport, and inflammatory responses. Imbalances lead to lipid accumulation and metabolic disorders.	Nuclear agonists: PPAR agonists (e.g., fenofibrate, pioglitazone). Potent lipid-lowering drugs: statins, PCSK9 inhibitors (e.g., evolocumab, which dramatically lowers LDL-C).
Interactions between the RAAS and lipid metabolism	Ang II, AT1R, NADPH oxidase, SREBP-1	RAAS overactivation leads to vasoconstriction, oxidative stress, and inflammation, and directly promotes lipid synthesis in a vicious cycle.	RAAS inhibitors: ACEI/ARB (e.g., ramipril, chlorosartan), the cornerstone of antihypertensive and cardiovascular protection. Combination therapy: RAAS inhibitors + statins/PCSK9 inhibitors for synergistic effect.
AMPK/SIRT1 signaling pathway	AMPK, SIRT1, eNOS, NF-κB, mTOR	Core regulator of cellular energy and metabolic homeostasis. Inhibition of function leads to metabolic disorders, oxidative stress, and inflammation.	Pathway activators: AMPK activators (e.g., metformin, AICAR), SIRT1 activators (e.g., resveratrol), with multiple benefits such as antihypertensive, lipid-modulating, and anti-inflammatory.

### 4.1 Endothelial function and NO signaling pathways

Endothelial dysfunction is a central component of the synergistic interaction between hypertension and dyslipidemia, with key mechanisms including eNOS uncoupling, reduced NO bioavailability, and enhanced oxidative stress. Ox-LDL triggers oxidative stress in endothelial cells. This stress disrupts the dimerization of eNOS and decreases the bioavailability of BH4. These changes lead to eNOS uncoupling. As a result, NO production is markedly reduced (30). This reduction in NO bioavailability leads to enhanced vascular smooth muscle contraction, increased vascular resistance, and thus promotes the development and maintenance of hypertension. Additionally, ox-LDL-mediated endothelial dysfunction promotes the expression of inflammatory mediators and adhesion molecules, enhancing inflammatory responses and remodeling of the vascular wall, further exacerbating vascular stiffness and blood pressure elevation (19, 59). Within this pathway, eNOS activators and antioxidants emerge as promising therapeutic targets. These substances enhance the stability of eNOS dimers, promote NO synthesis, and alleviate oxidative stress. This helps improve endothelial dysfunction and reduce vascular resistance. In turn, these effects may alleviate the pathological state of hypertension. Therefore, elucidating the molecular mechanisms by which dyslipidemia leads to endothelial dysfunction is of great significance for developing novel, precision-targeted therapeutic strategies for hypertension.

### 4.2 Inflammatory signaling pathways (NF-κB, NLRP3 inflammasome)

Dyslipidemia plays a key pathological role in the onset and progression of hypertension by activating inflammation-related signaling pathways, particularly the NF-κB and NLRP3 inflammasome pathways. Oxidized LDL and free fatty acids can significantly activate the NF-κB pathway. This activation prompts vascular endothelial cells and vascular smooth muscle cells to synthesize various pro-inflammatory factors (e.g., TNF-α, IL-1β, and IL-6) as well as adhesion molecules (e.g., VCAM-1 and ICAM-1). In turn, this promotes the formation of a local inflammatory microenvironment and immune cell infiltration, which ultimately leads to chronic vascular inflammation and dysfunction (60). At the same time, lipid deposition stimulates the assembly and activation of NLRP3 inflammasomes. This process mediates the caspase-1-dependent maturation and secretion of IL-1β, which further exacerbates inflammatory reactions in the vascular wall and promotes its remodeling processes (61). The aforementioned inflammatory response not only weakens vascular compliance and increases peripheral resistance but also disrupts the RAAS and SNS, exacerbating blood pressure dysregulation. Current research has demonstrated that inhibitors targeting the NF-κB signaling pathway (e.g., Bay 11-7082) or NLRP3 inflammasome-specific blockers (e.g., MCC950) can effectively reduce vascular inflammation and lower elevated arterial blood pressure in animal models, suggesting potential therapeutic applications (62). Therefore, inflammatory pathways provide an important pathogenic bridge between hypertension and dyslipidemia, and precise intervention targeting these pathways may become a new strategy for the treatment of hypertension in the future.

### 4.3 Lipid metabolism regulatory pathways (PPARs, LXR, and SREBP-1c)

Imbalances in lipid metabolism regulatory pathways, notably those mediated by nuclear receptors (PPARs, LXR) and transcription factors (SREBP-1c), are recognized as a core pathogenic mechanism in the interaction between hypertension and dyslipidemia. Members of the PPAR family, particularly PPAR*α* and PPAR*γ*, play a key role in regulating fatty acid oxidation, triglyceride metabolism, and anti-inflammatory responses. Studies have shown that PPAR*α* agonists not only effectively lower plasma triglyceride levels and improve lipid profiles but also significantly improve vascular function and blood pressure control by downregulating the expression of inflammatory factors and inhibiting the proliferation of vascular smooth muscle cells (63). PPAR*γ* agonists can help lower blood pressure through two key pathways to synergistically regulate vascular tone and reverse arterial stiffness. One is the endothelial pathway: it induces RBP7, which enhances the antioxidant state and promotes NO synthesis. The other is the smooth muscle pathway: it induces RhoBTB1-CUL3, which ubiquitinates and degrades PDE5. This process maintains cGMP levels and enhances the NO response, ultimately contributing to blood pressure reduction (64). On the other hand, liver X receptors (LXR) play a central role in regulating cholesterol efflux and reverse transport as cholesterol-sensing transcription factors. When activated, they boost the production of transport proteins like ABCA1. ABCA1 helps move cholesterol out of cells and onto apolipoprotein AI (apoAI). This process clears cholesterol from endothelial cells and reduces cholesterol buildup in arteries, thereby slowing the development of atherosclerosis (65). Sterol regulatory element-binding protein SREBP-1c, when abnormally activated, boosts the expression of lipid synthesis enzymes like FASN and SCD1. As a result, lipids accumulate locally in the liver and blood vessels. This accumulation worsens metabolic disorders and impairs vascular function (66). Thus, targeting PPARs and LXR to regulate lipid metabolism or inhibiting the SREBP-1c pathway to limit lipid synthesis has become an important therapeutic strategy for intervening in hypertension accompanied by dyslipidemia. This approach has demonstrated significant antihypertensive and vasoprotective effects in multiple animal and clinical studies.

### 4.4 Interactions between the RAAS and lipid metabolism

The RAAS plays a central role in blood pressure regulation and has a close bidirectional regulatory relationship with dyslipidemia, forming an important pathological basis for the synergistic progression of hypertension and dyslipidemia. Ang II is a key effector molecule of the RAAS. It directly mediates vascular smooth muscle contraction, which in turn increases peripheral resistance. Furthermore, Ang II activates NADPH oxidases (Nox), resulting in a significant increase in oxidative stress levels. These effects of Ang II are supported by research. This damages vascular endothelial cells, reduces NO bioavailability, and promotes the continued progression of endothelial dysfunction (67). In addition, Ang II activates SREBP-1 through the AT1R-PKC pathway, upregulates the expression of lipid synthesis enzymes such as FASN and ACC, enhances local lipid synthesis in the liver and blood vessels, and leads to lipid accumulation and metabolic disorders (68). At the same time, RAAS activation is closely related to inflammatory responses. It activates the NF-κB signaling pathway and promotes the release of proinflammatory factors such as IL-6 and TNF-α. This process forms a chronic low-grade inflammatory microenvironment that further aggravates atherosclerosis and hypertension (69). ACEIs and ARBs are key intervention strategies. They effectively block the production or action of Ang II, thereby lowering blood pressure. Additionally, these agents exert multiple beneficial effects by improving dyslipidemia, inhibiting inflammation, and counteracting oxidative stress (70). Recent studies offer new insights into the clinical use of RAAS-blocking drugs in combination with lipid-lowering drugs. This combination works synergistically to regulate blood pressure and blood lipids, thereby optimizing cardiovascular protection (71). Therefore, multi-pathway intervention targeting RAAS provides a theoretical basis and a new perspective for precision treatment in patients with hypertension and dyslipidemia.

### 4.5 AMPK/SIRT1 signaling pathway

The AMPK/SIRT1 signaling pathway, a pivotal regulatory hub coordinating energy metabolism and vascular homeostasis, exerts synergistic pleiotropic effects in the pathogenesis of hypertension-dyslipidemia syndrome. AMPK functions as a cellular energy sensor that is activated under conditions of cellular energy stress. Upon activation, AMPK promotes fatty acid oxidation (FAO) while inhibiting lipid synthesis. Furthermore, it upregulates the expression and activity of SIRT1, thereby forming a positive regulatory loop (72). SIRT1 enhances eNOS activity by deacetylating it directly or indirectly, thereby increasing phosphorylation at key activation sites (e.g., Ser1177). Additionally, SIRT1 suppresses the NF-κB pathway, reducing NOX-derived ROS production. These combined effects mitigate oxidative stress, preserve nitric oxide bioavailability, and significantly improve vascular endothelial function and blood pressure homeostasis (73). Moreover, the AMPK/SIRT1 axis downregulates lipid synthase expression by inhibiting the transcriptional activity of the mTORC1 complex and SREBP-1c, in turn reversing dyslipidemia and alleviating the burden of atherosclerosis (74). Research also shows that the AMPK/SIRT1 pathway reduces the levels of inflammatory factors (e.g., IL-6 and TNF-α) by inhibiting NF-κB activation, alleviating chronic low-grade inflammation, and providing important protection against the oxidative damage caused by hypertension and dyslipidemia (75). Numerous animal experiments and preclinical studies have yielded positive results. AMPK pathway activators, such as AICAR and metformin, as well as SIRT1 activators like resveratrol, show notable effects in lowering blood pressure, regulating lipids, and reducing inflammation. These findings offer a theoretical foundation for integrated precision intervention on metabolism and blood vessels and identify new drug targets for this purpose (76, 77). However, the specificity and clinical translational efficacy of these activators still need to be validated by larger-scale clinical trials.

## 5 Advances in targeted therapy research

**Figure 4:**
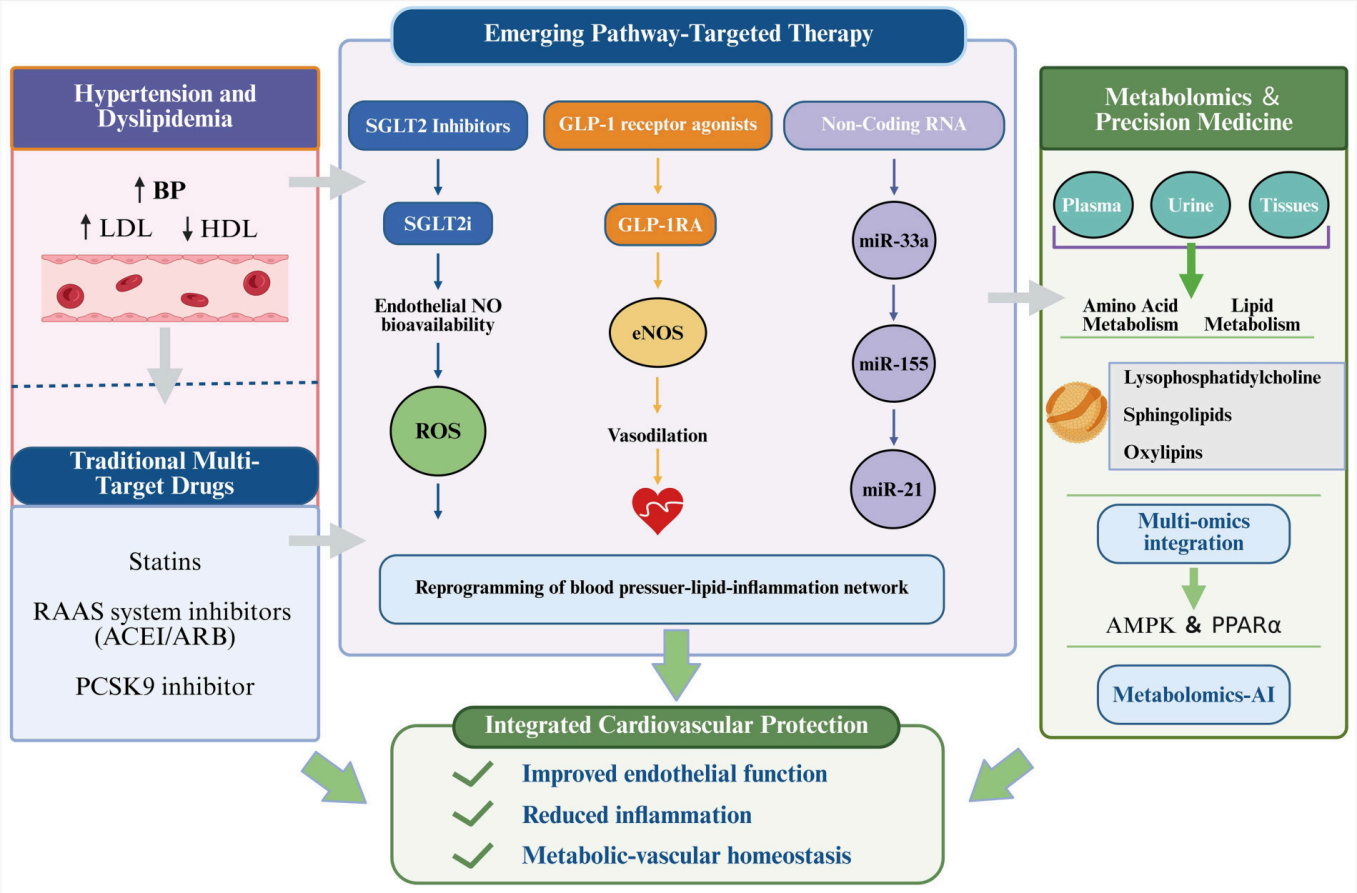
The therapeutic strategies targeting hypertension and dyslipidemia include conventional treatments, emerging pathway-targeted interventions, and metabolomics-based precision medicine.

With the deepening of the understanding of the complex pathogenesis of CVD, single-target therapies are facing challenges due to limited efficacy, easy drug resistance, and difficulty in blocking the pathology of multiple pathways. Currently, the focus of research is shifting from “single-target intervention” to “multi-target synergistic intervention” to synergistically regulate multiple pathogenic links, reshape metabolic-vascular homeostasis, and enhance cardiovascular protection. The synergistic pathogenesis of hypertension and dyslipidemia, which are frequently coexisting CVD risk factors, involves multiple mechanisms. This provides a theoretical basis for multi-targeted combination therapy. Therefore, this section systematically reviews the recent advances in multi-target drug combinations, emerging targeted pathways, and metabolomics-guided precision interventions (Figure 4).

### 5.1 Multi-target drugs and combination therapy strategies

In patients with coexisting hypertension and dyslipidemia, traditional single-target interventions often fail to effectively curb the progression of cardiovascular events. In recent years, multi-target drug combination therapy strategies have become a key focus of clinical and basic research. One notable combination is RAAS inhibitors (including ACEI and ARB) paired with statins. This specific drug combination has shown remarkable cardiovascular protective effects. ACEI/ARB work by blocking the vasoconstriction, inflammation, and oxidative stress responses mediated by angiotensin II, thereby improving vascular function and delaying arterial remodeling (78). Statins, on the other hand, lower LDL-C levels by inhibiting HMG-CoA reductase and have multiple effects, including anti-inflammatory, plaque stabilization, and improved endothelial function (79). The two have synergistic mechanisms in terms of endothelial protection and slowing the progression of atherosclerosis. Clinical studies have confirmed that their combined use can significantly reduce the risk of cardiovascular events (80). The Systolic Blood Pressure Intervention Trial provided clear evidence by comparing two blood pressure targets: the standard target of systolic blood pressure <140 mmHg and the intensive target of systolic blood pressure <120 mmHg. Intensive blood pressure control showed significant benefits: it reduced the risk of major cardiovascular events by roughly 25% and lowered all-cause mortality by 27%. These benefits were especially notable in high-risk populations with dyslipidemia (81). This result reinforces the central role of strict blood pressure control in high-risk populations and provides high-level evidence-based support for the use of RAAS inhibitors in combination therapy.

Moreover, the clinical use of PCSK9 inhibitors has further refined targeted strategies for patients with severe hypercholesterolemia. PCSK9 inhibitors can enhance the circulating expression of LDL receptors, significantly reducing LDL-C levels. When used in combination with antihypertensive drugs, they not only optimize lipid control but also reduce the formation of ox-LDL by significantly lowering LDL-C levels, thereby alleviating endothelial oxidative damage and improving vascular dilatory function (82). The FOURIER trial further demonstrated that the addition of the PCSK9 inhibitor evolocumab to statin therapy resulted in a further reduction in LDL-C levels of approximately 59% and a significant 15% reduction in the risk of major cardiovascular events, with a favorable safety profile (83). Future research may further explore the potential synergistic effects of combining PCSK9 inhibitors with ACE inhibitors or calcium channel blockers in reducing inflammatory markers and vascular stiffness, providing new intervention strategies for multi-target treatment approaches in high-risk patients.

### 5.2 Emerging therapeutic targets and strategies

In addition to traditional multi-target drugs, targeted therapies aimed at emerging pathways are emerging as a research hotspot. In recent years, treatment strategies for the co-morbidity of hypertension and dyslipidemia have shifted from intervening in traditional pathways to targeting novel molecular pathways. Clinical studies have demonstrated that sodium-glucose cotransporter 2 inhibitors (SGLT2i) and glucagon-like peptide-1 receptor agonists (GLP-1RA) represent breakthrough advancements in the treatment of cardiovascular and renal metabolic diseases, exhibiting dual protective effects on the cardiovascular system (84, 85). SGLT2 inhibitors exert their cardioprotective effects by promoting endothelial NO bioavailability and reducing inflammation and reactive ROS in cardiac endothelial cells. Additionally, some studies suggest that SGLT2 inhibitors may also exert endothelial protective effects through pathways such as influencing endothelial cell ion transport (e.g., inhibiting NHE1 and regulating NCX). The specific mechanisms underlying these effects are currently under further investigation (86). GLP-1RAs effectively regulate blood glucose levels. Beyond this primary action, they exert multi-target effects, including anti-inflammatory properties, improved lipid profiles, and enhanced endothelium-dependent vasodilation. Some studies have shown that they may mediate cardiovascular protection by inhibiting the NF-κB signaling pathway and improving eNOS function in chronic low-grade inflammatory processes (87).

At the same time, non-coding RNAs such as miRNAs are increasingly recognized as upstream factors regulating disease phenotypes and key nodes connecting environmental stimuli, signaling pathways, and phenotypic changes. For example, miR-33a influences cholesterol efflux and HDL production by targeting ABCA1, thereby participating in the formation of dyslipidemia (88). miR-155 is a typical TNF-induced miRNA. It plays an important role in regulating immune adhesion and vascular inflammation. Under shear stress and inflammatory stimulation, miR-21 regulates the expression of molecules like eNOS and ET-1, participating in the NO signaling pathway and contributing to endothelial dysfunction (89). With their enhanced specificity and multi-pathway regulatory capabilities, these molecules represent a promising avenue for advancing precision treatment strategies beyond traditional drugs. Specific targeted intervention through antisense oligonucleotides or miRNA mimics can reshape the blood pressure-lipid-inflammation network at the systemic level (90). Despite their promising therapeutic potential, these molecules face critical translational challenges, including limited delivery efficiency, off-target effects, and unresolved long-term safety concerns.

### 5.3 Metabolomics-driven precision intervention strategies

With the development of systems biology, metabolomics has become one of the key technologies for elucidating the interaction mechanisms between hypertension and dyslipidemia and identifying target intervention points. Through high-throughput analysis of small molecule metabolites in plasma, urine, and tissues, metabolomics has revealed metabolic pathway disorders associated with hypertension, including disruptions in amino acid metabolism, dyslipidemia, and abnormal fatty acid β-oxidation (91). It can also identify lipid subtypes closely related to atherosclerosis risk, for instance, lysophosphatidylcholine, sphingolipids, and oxylipins (92, 93). These metabolites, as potential biomarkers, not only aid in the early identification of high-risk patients but can also be used to guide the development of personalized intervention strategies. Meanwhile, changes in lipid profiles can also reflect individual differences in response to lipid-lowering or antihypertensive drugs, suggesting that metabolomics data can be used for treatment sensitivity assessment and risk stratification (94).

More importantly, the integrated application of metabolomics within the framework of precision medicine has driven a shift from “disease cause identification” to “intervention decision-making.” Researchers, by using transcriptomics, epigenomics, and microbiomics for multi-omics integration, can build metabolic network interference models. They also identify key target pathways, including the AMPK pathway and the PPAR*α* regulatory network. This work provides a basis for developing multi-pathway synergistic intervention strategies (95, 96). At the same time, machine learning and artificial intelligence (AI) technologies can be used to construct personalized metabolic feature prediction models, enabling dynamic optimization of treatment plans and response monitoring. In the future, precision interventions driven by metabolomics data are expected to play a core supporting role in the early screening, classification, monitoring, and treatment of hypertension and dyslipidemia (97).

## 6 Future perspectives

Although significant progress has been made in understanding the pathogenesis of CVD and the synergistic interplay between hypertension and dyslipidemia, critical gaps remain regarding their spatiotemporal dynamics, tissue microenvironment heterogeneity, and inter-individual variability. Future research should prioritize the integration of multi-omics technologies like spatial transcriptomics and single-cell metabolomics to map the real-time molecular interactions and metabolic exchange among different vascular cell types (e.g., endothelial cells, smooth muscle cells, and immune cells) under pathological stimuli. Engineered organoids and organ-on-a-chip models (e.g., vascular-immune co-culture systems) will be instrumental in precisely simulating the human-specific microenvironment of coexisting hypertension and dyslipidemia, thereby revealing dynamic intercellular patterns, including metabolite transfer, signaling crosstalk, and phenotypic transformation. Coupled with AI-driven multimodal data fusion, these approaches can help construct a metabolic-phenotype-based risk-stratification and early-warning model for CVD. In terms of intervention, moving beyond traditional single-target therapies is essential. Future strategies should focus on developing spatiotemporally precise drug delivery systems (such as exosome-loaded drugs and responsive nanoparticles) capable of synergistically modulating key regulatory networks such as the AMPK/SIRT1-PPAR-LXR. Moreover, the therapeutic potential of CRISPR/Cas9-mediated gene editing and non-coding RNA (e.g., miR-33a/155) should be explored to reverse persistent endothelial dysfunction and pathological metabolic reprogramming. Prospective large-scale validation of novel biomarker panels, such as sphingolipid profiles and oxidized lipid metabolites, will be crucial for guiding personalized treatment regimens, including a rational combination of SGLT2 inhibitors, GLP-1 receptor agonists, and PCSK9 inhibitors. Ultimately, these advances aim to shift the clinical paradigm from reactive disease treatment toward active restoration of metabolic-vascular homeostasis. In summary, future efforts should elucidate the spatiotemporal dynamics and individual heterogeneity underlying the hypertension-dyslipidemia synergy to develop a multi-target, mechanism-based intervention. The integration of multi-omics profiling, AI-driven risk prediction, and phenotype-guided therapy will be key to implementing precision medicine in CVD care—transitioning from conventional intervention to homeostasis maintenance and ushering in a new era of precision prevention and treatment for cardiovascular metabolic diseases.

## 7 Conclusion

Hypertension and dyslipidemia represent the two most significant risk factors for CVD. Rather than acting in isolation, they engage in a complex molecular network of interactions. Their synergistic effects drive vascular endothelial dysfunction, oxidative stress, and immune homeostatic imbalance through multiple interconnected mechanisms, including dysregulation of the RAAS/PPAR axis, inhibition of AMPK/SIRT1 signaling, and sustained activation of chronic inflammation. Collectively, these processes accelerate atherosclerosis and promote end-organ cardiovascular damage. A deep understanding of the synergistic mechanism linking hypertension and dyslipidemia holds substantial clinical and translational importance. First, it supports a shift in CVD prevention from single-target intervention toward multi-target, network-based, and individualized precision therapeutic strategies. Second, it provides a strong theoretical foundation for developing novel combination therapies and optimizing existing clinical regimens. Ultimately, this knowledge will help transition the management paradigm from the traditional “disease treatment” to proactive “metabolic-vascular homeostasis restoration”, thereby improving the overall prevention and treatment landscape for CVD.
